# Conserved NT5C2 links context-specific behaviors with psychiatric and metabolic risk

**DOI:** 10.1186/s12993-025-00314-w

**Published:** 2026-01-06

**Authors:** Thiago C. Moulin, Iván Aldavero-Muñoz, Michael J. Williams, Helgi B. Schiöth

**Affiliations:** 1 Department of Surgical Sciences, Uppsala University, Uppsala, Sweden; 2 Department of Pharmaceutical Biosciences, Uppsala University, Uppsala, Sweden; 3 Universidad Católica de Ávila, Ávila, Spain; 4 Laboratory of Pharmaceutical Pharmacology, Latvian Institute of Organic Synthesis, Riga, Latvia

## Abstract

**Background:**

The cytosolic 5′-nucleotidase II (NT5C2) enzyme has been implicated in both psychiatric disorders and metabolic traits, but whether these associations reflect a shared biological basis remains unclear. Here we combined cross-species approaches to investigate how reduced NT5C2 function shapes behavior.

**Results:**

In *Drosophila melanogaster*, neuronal knockdown of the ortholog dNT5B increased activity around light-dark transitions, reduced sleep fragmentation, and selectively suppressed food intake under satiated conditions. Moreover, analysis of mouse phenotyping data revealed that whole-body Nt5c2 knockout alters locomotor activity, sensorimotor gating, and anxiety-related behaviors. Finally, human variant-trait associations showed reproducible enrichment in both metabolic domains, including body composition and BMI, and neuro-psychiatric outcomes such as schizophrenia, smoking, and anxiety.

**Conclusions:**

Together, these phenotypic findings indicate that NT5C2 is a conserved neuro-metabolic regulator, linking energy-related pathways to specific behavioral dimensions that may underlie its pleiotropic impact on psychiatric and metabolic risk.

## Background

The NT5C2 gene encodes the cytosolic 5′-nucleotidase II (cN-II) enzyme, a purine phosphatase that hydrolyzes inosine monophosphate (IMP), guanosine monophosphate (GMP), and, less efficiently, adenosine monophosphate (AMP) [1]. cN-II activity is allosterically stimulated by ATP and suppressed during metabolic energy depletion, positioning it upstream of AMP-activated protein kinase (AMPK) signaling, and thus affecting energy homeostasis and protein synthesis pathways [2, 3]. In the nervous system, NT5C2 expression is enriched in neurons and higher during human neurodevelopment, consistent with roles in brain energy regulation and translational control [4]. NT5C2 is highly conserved across evolution in eukaryotes [5]. For instance, the *Drosophila* ortholog dNT5B (CG32549) shares ~ 80% sequence similarity with human NT5C2 [4], and the *C. elegans* gene Y71H10B.1 encodes a homologous enzyme with similar roles [6]. All these orthologs retain the same domain architecture and catalytic machinery for the hydrolysis of 5′-monophosphate purine nucleotides (AMP, IMP, GMP) to their nucleosides, thereby modulating purine pools [7, 8]. Regulatory mechanisms are likewise conserved. Its allosterically activation by ATP/ADP and inhibition by inorganic phosphate has been seen in the human and *Drosophila* enzymes [7, 9].

In humans, common variant studies repeatedly implicate NT5C2 in neuropsychiatric risk. GWAS identified risk loci at NT5C2 (e.g., rs11191580), with associations spanning schizophrenia [10, 11], bipolar disorder [12], autism spectrum disorder [13], insomnia [14] and affective disorders [15]. Such risk alleles are associated with reduced NT5C2 expression in fetal and adult brain, pointing to a loss-of-function mechanism [16]. Moreover, rare biallelic NT5C2 loss-of-function causes SPG45, a complex neurodevelopmental syndrome with spastic paraplegia, intellectual disability, thin corpus callosum, and ADHD-like features, strengthening NT5C2’s importance for nervous system development [17, 18]. Genetic comorbidity work further flagged the NT5C2 schizophrenia-associated SNP in Parkinson’s disease, suggesting pathway overlap across psychiatric and neurodegenerative phenotypes [19].

Independent lines of evidence also link NT5C2 to metabolic and body weight regulation. In humans, NT5C2 rs11191580 has been shown to influence body mass index (BMI) in adults [20]. Other NT5C2 polymorphisms are associated with reduced abdominal fat, indicating effects on adiposity distribution [21], and are suggested to mediate the association between BMI and major depressive disorder [22]. In mice, Nt5c2 knockout confers protection against diet-induced obesity and insulin resistance [23]. Additionally, consistent with its high energy-sensing function, adipose tissue from Nt5c2 knockout mice shows increased AMPK pathway readouts under stimulation [23]. Cross-phyla functional genomics echo these findings, as RNAi of the *C. elegans* ortholog reduces fat storage without developmental delay and prevents diet-induced obesity in a high-fructose model [6]. Overall, this cross-species evidence suggest NT5C2 normally dampens cellular AMP signaling and reducing NT5C2 activity shifts energy homeostasis towards insulin sensitivity and reduced fat storage.

Mechanistic studies converge on AMPK-mTOR-translation coupling. Cytosolic 5′-nucleotidases regulate purine nucleotide balance and thereby tune the AMP cycle [3]. In human myotubes, NT5C2 silencing elevates the AMP: ATP ratio, enhances AMPK phosphorylation at Thr172, and increases phosphorylation of its substrate acetyl-CoA carboxylase (ACC) [2]. However, in contracting skeletal muscle of NT5C2- or NT5C1A-deficient mice, AMPK activity was not potentiated, suggesting that nucleotide fluxes during exercise override nucleotidase control [24]. In the nervous system, NT5C2 knockdown in human neural progenitor cells increased AMPK expression and phosphorylation, while also unexpectedly raising phosphorylation of ribosomal protein S6 (RPS6), a marker of mTORC1 activity, with transcriptional signatures indicating altered translational regulation [16]. Conversely, overexpression of NT5C2 in HEK293T cells decreased AMPK phosphorylation and was associated with enhanced RPS6 phosphorylation, highlighting context-specific effects [16]. In human lung carcinoma A549 cells, NT5C2 knockdown caused a dramatic reduction in protein synthesis without modifying AMPK activity, likely due to LKB1 inactivation in this cell line [25, 26]. Together, these findings indicate that NT5C2 modulates the AMPK–mTOR axis in a tissue-dependent manner, with consequences for energy sensing, protein synthesis, and neuronal network function [27–29].

Moreover, in the presence of high energy charge, NT5C2 activity promotes hydrolysis of excess nucleotides, whereas under low energy charge, AMP accumulation can either activate AMPK or be degraded by NT5C1 to adenosine, initiating purinergic signaling [30]. Extracellular adenosine, via A1, A2A, A2B, and A3 receptors, modulates synaptic fine-tuning and neuronal network coordination, while also regulating blood flow, inflammation, and immune responses [31–33]. Adding further brain-specific relevance, NT5C2 physically interacts with the schizophrenia-associated protein ZNF804A in cortical neurons, where both proteins influence each other’s subcellular distribution, suggesting a shared pathway linking energy regulation, synaptic structure, and psychiatric risk [34]. The ability of NT5C2 to modulate these pathways highlights its central role in coordinating metabolic, translational, and neuromodulatory processes.

Additionally, the *Drosophila* ortholog dNT5B has been used to probe conserved roles of this gene family in vivo, as this model provides a powerful tool for dissecting the molecular underpinnings of behavior [35]. For instance, a recent study has found high levels dNT5B expression in the fly brain at later pupal and adult stages [8]. Neuronal knockdown of dNT5B has yielded somewhat divergent outcomes across studies. Duarte et al. reported that pan-neuronal or ubiquitous knockdown using the ELAV or ACT5C drivers led to pronounced impairments in climbing ability, a classical assay of fly motility behavior, whereas gut-specific knockdown produced no deficit [4]. In contrast, Singgih et al. found that pan-neuronal knockdown using the nSyb driver did not affect habituation learning in the light-off jump reflex paradigm and produced only mild alterations in sleep and activity, with no major changes in synaptic morphology at the neuromuscular junction [8]. These contrasting results may reflect differences in behavioral paradigms (negative geotaxis versus startle habituation), in the neuronal drivers employed (ELAV versus nSyb), or in the extent of knockdown across neuronal populations. Together, the available evidence suggests that dNT5B plays a neuronal role in regulating motor-related behaviors in flies, but the precise nature and robustness of these effects remain to be clarified, and may depend on circuit-specific requirements and experimental approach.

Building on this work, we used the Gal4-UAS system to selectively knock down dNT5B in neurons and systematically assessed activity and sleep phenotypes, while also interrogating feeding behavior for the first time. Expanding the behavioral scope and including different fly lines allowed us to obtain a more comprehensive view of how reduced NT5C2 function influences both motor and metabolic dimensions in the fly. Moreover, we sought to create a cohesive cross-species phenotypic characterization connecting NT5C2’s behavioral effects to its psychiatric and metabolic genetic associations. We combined insights from *Drosophila* behavioral assays with mouse phenotype data and human genetic evidence, examining whether reduced NT5C2 function shows conserved, context-dependent alterations in behavioral reactivity and feeding regulation, features that mirror pleiotropic associations spanning psychiatric and metabolic domains.

## Methods

### Fly Husbandry, genotypes and handling

Fly stocks were maintained at 25 °C with ~ 60% relative humidity in a 12:12 h light: dark cycle (lights on at 08:00). Unless otherwise stated, flies were reared on Jazz-Mix™ Drosophila food (Thermo Fisher Scientific, Göteborg, Sweden) supplemented with 8.3% yeast. Stocks were maintained in bottles with ≤ 70 flies per culture, flipped weekly into fresh food, and discarded after one month. The following strains were obtained from the Bloomington Drosophila Stock Center (BDSC, Bloomington, IN, USA): (i) elav^C155^-GAL4, a pan-neuronal driver (BDSC #458; genotype: *P{w[+ mW.hs] = GawB}elav[C155]*); (ii) UAS-dNT5B RNAi (BDSC #57238; genotype: *y* [1] sc[*] v [1] *sev* [21]; *P{y[+ t7.7] v[+ t1.8] = TRiP.HMC04624}attP40*); (iii) w^1118^, used as a wild-type control. Knockdown flies were generated by crossing virgin females elav^C155^-GAL4 (X-linked) with males of the UAS-RNAi line, yielding neuronal knockdown in the F1 progeny. Two control groups were used: (i) elav^C155^-GAL4 females crossed with w^1118^ males, and (ii) w^1118^ females crossed with UAS males. Crosses were typically set with ~ 35 virgin females and ~ 15 males per bottle.

### Feeding behavior

Feeding behavior was quantified using the FlyPad system, a capacitive-based platform that records proboscis-food interactions [36]. Individual flies were transferred by mouth aspiration into arenas containing two food channels, one filled with 4–5 µL of standard liquid food and the other left empty. The primary outcome measure was the number of sips, defined as a single proboscis-food interaction, which strongly correlates with actual food intake volume [36]. As a secondary metric, we analyzed the mean duration of feeding bouts (food-approach episodes), reflecting the average feeding time per approach. Both measures have been validated as reliable proxies of food consumption. Assays were conducted for one hour between 08:00 and 11:00, with control and knockdown groups tested in parallel under identical conditions. Both fed and starved flies were assayed; for the starvation condition, flies were deprived of food for ~ 16 h with water available. Each group included at least 8 flies per assay. Flies that remained completely immobile for the duration of the assay were excluded from analysis. The experiments were performed using the 2018 FlyPad version.

### General activity and sleep

General locomotor activity and sleep patterns were evaluated using the Drosophila Activity Monitor System (DAMS; TriKinetics Inc., Waltham, MA, USA), following established protocols [37]. Flies were briefly anesthetized with CO₂ and individually transferred into horizontal plastic tubes sealed at one end with standard fly food and at the other with a cotton plug to allow ventilation. An infrared beam at the midpoint of each tube recorded every crossing as an activity count. Activity was recorded continuously over 3 days under a 12:12 h light: dark cycle, starting at lights-on. To allow acclimation, the first 24 h of data were excluded from analysis. Raw data were exported as CSV files using DamFileScan (TriKinetics). Subsequent analysis of sleep and circadian activity parameters was performed with the Sleep and Circadian Analysis MATLAB Program (SCAMP, Vecsey Lab). Locomotor activity was defined as the number of infrared beam crossings per unit time. Sleep was defined as periods of uninterrupted inactivity lasting ≥ 5 min, a standard operational definition in *Drosophila* sleep research. Flies that remained completely immobile for the duration of the assay were considered dead and excluded from analysis.

### Statistical analysis

All data were analyzed using GraphPad Prism (v. 10.4; GraphPad Software, San Diego, CA, USA). For comparisons of more than two groups, one-way ANOVA followed by Tukey’s post hoc test was used. When repeated measures or temporal data were analyzed, two-way ANOVA was applied with Holm–Šidák post hoc tests for multiple comparisons. Data are presented as mean ± SEM, and significance thresholds were set at *p* < 0.05. Post-hoc sensitivity analysis were performed using the G*Power software (v. 3.1.9.7) [38].

### Mouse phenotype and GWAS dataset analyses

Mouse knockout phenotypes for Nt5c2 were obtained from the International Mouse Phenotyping Consortium (IMPC, www.mousephenotype.org) [39], accessed in March 2020. The dataset included standardized behavioral and metabolic assays performed in both male and female Nt5c2 knockout and wild-type littermates. Among the assays available, we focused on those with significant genotype effects, including locomotor activity measured in the combined SHIRPA and dysmorphology screen, acoustic startle and pre-pulse inhibition (PPI), and the open field test. These assays assess, respectively, general locomotor behavior and reflexes, sensorimotor gating, and exploratory/anxiety-related behaviors. Experimental protocols and quality control procedures are standardized across IMPC centers. Summary-level data were extracted directly from the IMPC database.

To evaluate human genetic associations, we queried the NHGRI-EBI GWAS Catalog and the GWAS Atlas database [40], accessed in June 2020. At the time of access, the GWAS Atlas included data from 4155 GWAS across 2960 traits and 1488 electronic health record–derived PheWAS codes. Only associations surpassing the study-wide corrected significance threshold (*p* < 0.05 after multiple-testing correction) were retained for analysis. Traits were grouped into broader categories (metabolic, neuro-psychiatric, cardiovascular, immunological, and others) to facilitate interpretation.

## Results

### Neuronal dNT5B knockdown influences light-dark transition activity and sleep fragmentation

**Fig. 1:**
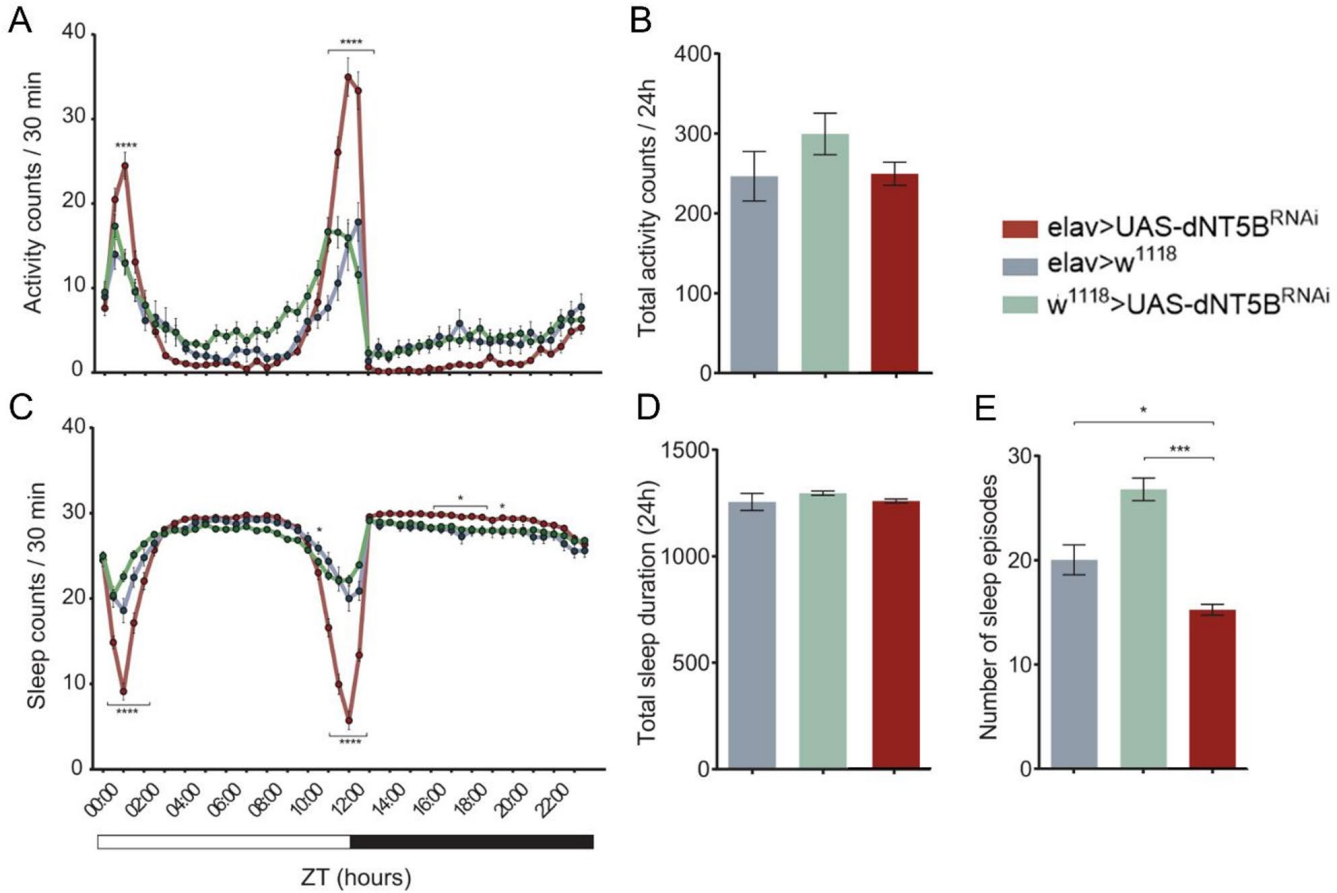
Neuronal dNT5B knockdown alters activity around light–dark transitions and reduces sleep fragmentation. **A** Locomotor activity counts (30 min bins) across 24 h under 12:12 h light–dark cycles. Knockdown flies (elav > UAS-dNT5B^RNAi^, red) displayed pronounced activity peaks after lights-on and lights-off times compared with controls (elav > w^1118^, blue; w^1118^ > UAS-dNT5B^RNAi^, green). **B** Total 24 h activity counts did not differ significantly between genotypes. **C** Sleep counts (30 min bins) showed reduced sleep during light–dark transitions in knockdown flies, accompanied by a significant compensatory increase in sleep during the dark phase. **D** Total sleep duration (24 h) was not significantly altered. **E** Knockdown flies exhibited significantly fewer sleep episodes, consistent with reduced sleep fragmentation. Two-way ANOVAs confirmed significant main effects of genotype, time, and their interaction (*p* < 0.0001) for both activity **(A)** and sleep counts **(C)**. Data are mean ± SEM. Sample sizes: elav > UAS-dNT5B^RNAi^ = 62, elav > w^1118^ = 34, w; w^1118^ > UAS-dNT5B^RNAi^ = 90. Panels A and C: two-way ANOVA with Holm–Sidak post hoc tests; Panels B, D, and E: one-way ANOVA with Tukey’s post hoc test. *p < 0.05, **p < 0.01, ***p < 0.001, ****p < 0.0001

To assess the role of dNT5B in neuronal regulation of rest-activity cycles, we monitored locomotor activity and sleep behavior in flies using the DAMS assay. Knockdown of dNT5B in neurons (elav > UAS-dNT5B^RNAi^) produced activity patterns broadly similar to controls (elav > w^1118^ and w^1118^ > UAS-dNT5B^RNAi^). However, two-way ANOVA revealed significant effects of genotype [F(2, 8784) = 18.75, *p* < 0.0001] and genotype × time interaction (F(94, 8784) = 9.846, *p* < 0.0001). These effects arose primarily from pronounced differences around light-dark transitions (Fig. 1A). Holm-Sidak post hoc tests showed that knockdown flies had significantly higher activity at ZT 00:30 compared to elav > w^1118^ (*p* = 0.018), and at ZT 01:00 they were significantly more active than both controls (*p* < 0.0001 for both comparisons), as well as during the ~ 1.5 h window around lights-off (ZT 11:30–12:30; *p* < 0.0001 for all time points compared to both controls). A modest trend toward reduced nighttime activity was also observed, although it did not reach significance. Despite these temporal differences, total 24 h activity counts did not differ across genotypes [one-way ANOVA; F(2, 183) = 1.518, *p* = 0.222] (Fig. 1B), suggesting that transition-related hyperactivity was offset by reduced nighttime activity.

Consistent with the activity data, two-way ANOVA on sleep counts revealed significant effects of genotype (F(2, 8784) = 41.44, *p* < 0.0001) and genotype × time interaction [F (94, 8784) = 28.65, *p* < 0.0001]. Holm-Sidak tests showed that knockdown flies exhibited reduced sleep during the hours surrounding lights-on and lights-off (ZT 00:30 − 02:00 and ZT 10:30 − 12:30; *p* < 0.0001 for all time points compared to both controls), mirroring the sharp increases in locomotor activity (Fig. 1C). Interestingly, during the dark phase, knockdown flies displayed a subtle but significant increase in sleep counts compared to controls (ZT 16:00–18:30 and ZT 19:30; *p* ranging from 0.006 to 0.0476), partially compensating for the transition-related losses. Accordingly, no significant genotype differences were detected in the total 24 h sleep duration [one-way ANOVA; F(2, 183) = 2.118, *p* = 0.123] (Fig. 1D). By contrast, analysis of sleep architecture revealed a marked reduction in sleep episode number in knockdown flies relative to both controls [one-way ANOVA; F(2, 183) = 36.17, *p* < 0.0001)] (Fig. 1E), indicating reduced sleep fragmentation and suggesting that neuronal dNT5B contributes to the temporal organization of sleep rather than overall sleep quantity.

### Neuronal dNT5B knockdown reduces food intake in satiated states

**Fig. 2:**
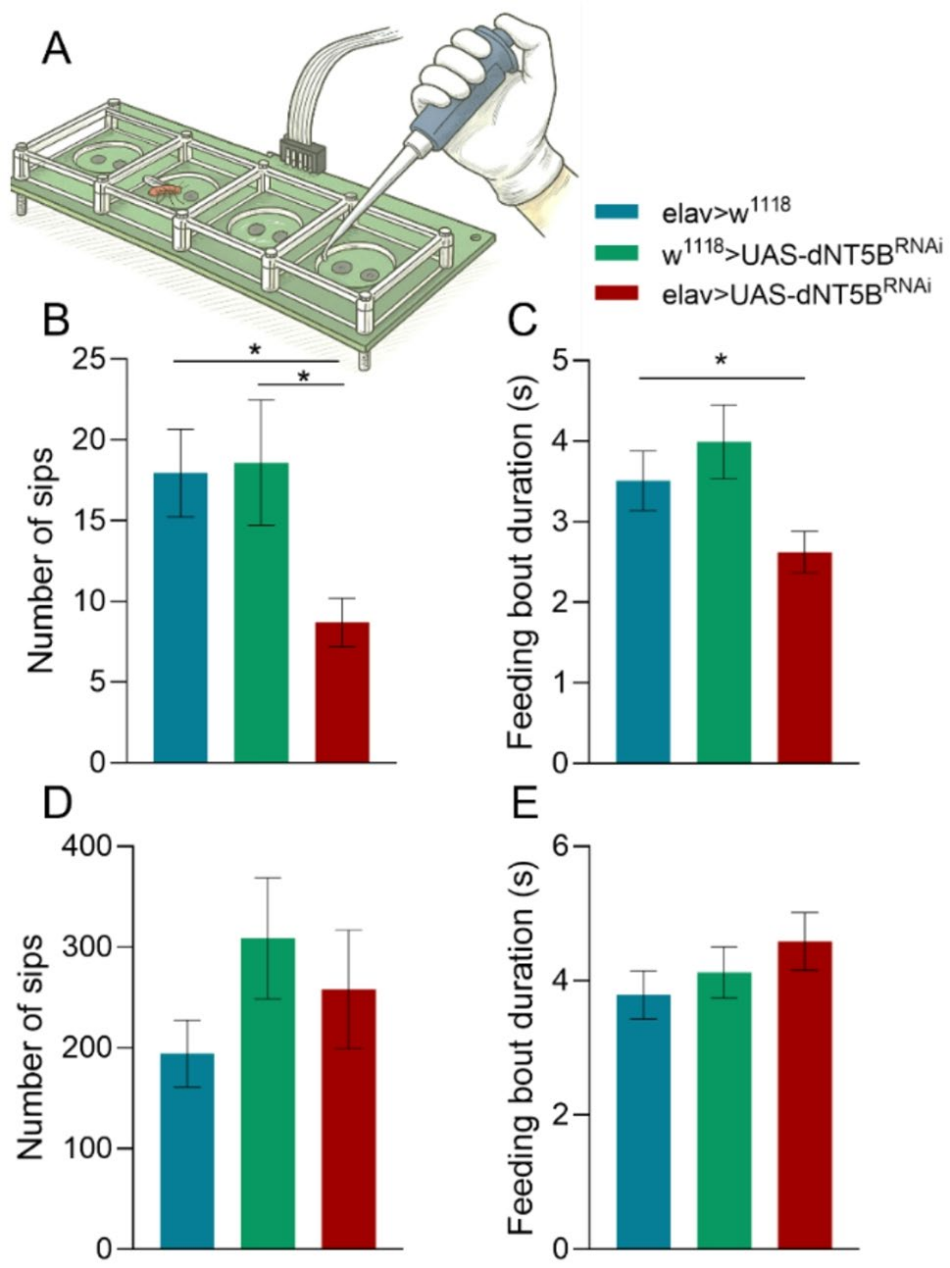
Neuronal dNT5B knockdown reduces food intake in satiated flies but not in starved flies. (**A**) Schematic of the FlyPad system, which detects proboscis-food interactions using capacitive sensors. **B** In satiated flies, knockdown (elav > UAS-dNT5B^RNAi^, red) resulted in significantly fewer sips compared to both controls (elav > w^1118^, blue; w^1118^ > UAS-dNT5B^RNAi^, green). **C** Mean feeding bout duration was also reduced in knockdown flies, reaching significance compared to w^1118^ > UAS-dNT5B^RNAi^. **D–E** Under starvation, no differences in sip number **(D)** or feeding bout duration **(E)** were observed across genotypes. Data are mean ± SEM. Sample sizes: satiated condition (**B**–**C**): elav > UAS-dNT5B^RNAi^ = 51, elav > w^1118^ = 61, w^1118^ > UAS-dNT5B^RNAi^ = 59; starved condition (**D**–**E**): elav > UAS-dNT5B^RNAi^ = 11, elav > w^1118^ = 10, w^1118^ > UAS-dNT5B^RNAi^ = 28. One-way ANOVA with Tukey’s post hoc test were performed in all panels. *p < 0.05

To determine whether neuronal dNT5B influences feeding behavior, we measured food interactions in the FlyPad system, which quantifies proboscis contacts with liquid food (Fig. 2A). When tested in the satiated state, knockdown flies (elav > UAS-dNT5B^RNAi^) displayed a marked reduction in food intake [one-way ANOVA; F (2, 168) = 3.27, *p* = 0.04]. Specifically, the number of sips over the assay period was significantly lower in knockdown flies relative to elav > w^1118^ (*p* = 0.044) and w^1118^ > UAS-dNT5B^RNAi^ (*p* = 0.0443) groups (Fig. 2B). Similarly, the mean duration of feeding bouts was reduced in knockdown flies [one-way ANOVA; F (2, 124) = 3.04, *p* = 0.05], reaching statistical significance compared with w^1118^ > UAS-dNT5B^RNAi^ (*p* = 0.044) (Fig. 2C). By contrast, when flies were food-deprived for ~ 16 h prior to testing, no differences were observed across genotypes. Both number of sips [F (2, 46) = 0.713, *p* = 0.495] and bout duration [F (2, 45) = 0.569, *p* = 0.57] were comparable between knockdown and control groups under starvation conditions (Figs. 2D-E). These findings indicate that neuronal dNT5B contributes to the regulation of feeding under conditions of satiety but does not constrain feeding behavior when hunger drives food intake, suggesting that dNT5B acts as a modulator of consumption rather than as an absolute requirement for feeding.

Importantly, as starvation trials and the transfer process resulted in substantial mortality, some groups ultimately exhibited reduced sample sizes. To evaluate the sensitivity of the assay under these constraints, we performed a post-hoc sensitivity analysis in G*Power 3.1. Using the final group sizes (*n* = 10–28) and an assumed power of 0.80, the analysis indicated that the design was sufficiently powered to detect effect sizes of approximately Cohen’s d ≈ 0.68. Effects of this magnitude fall within the moderate range according to Cohen’s conventional benchmarks [41]. Thus, while we retain adequate sensitivity to detect medium or larger effects, we cannot rule out the presence of smaller genotype-dependent differences in feeding under starvation conditions.

### Nt5c2 knockout alters PPI and anxiety phenotypes

**Fig. 3:**
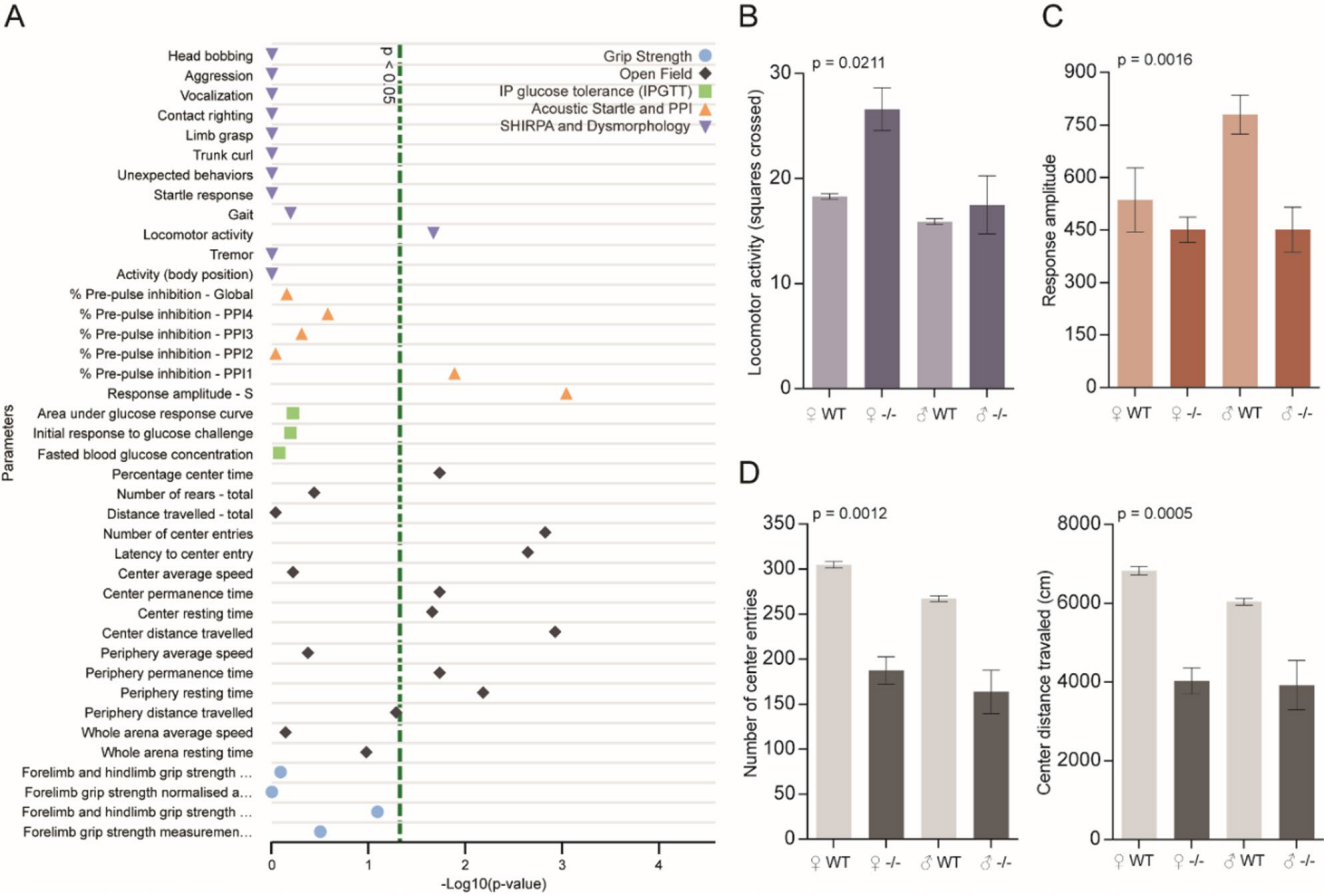
Nt5c2 knockout in mice alters locomotion, sensorimotor gating, and anxiety-related behaviors (IMPC dataset). **A** Significance plot of behavioral and metabolic outcomes from IMPC assays relevant to behavior or metabolism. The dashed line marks the *p* = 0.05 threshold. **B** Locomotor activity (squares crossed, SHIRPA and dysmorphology assays) was reduced in KO mice compared with WT, with effects most pronounced in females (two-way ANOVA, genotype effect *p* = 0.0211). **C** Response amplitude to acoustic startle was reduced in KO mice, particularly in males (*p* = 0.0016). PPI1 was also significant but not shown. **D** Open field behavior revealed reduced center entries (*p* = 0.0012) and center distance traveled (*p* = 0.0005) in KO mice. Other significant open field measures are not shown. Bars represent mean ± SEM, with WT groups shown in light color and KO groups in dark color. Sample sizes: (*B*) Female WT = 649, Female KO = 9, Male WT = 662, Male KO = 10; (**C**) Female WT = 7, Female KO = 9, Male WT = 10, Male KO = 10; (**D**) Female WT = 640, Female KO = 9, Male WT = 649, Male KO = 10

To extend our findings from *Drosophila* assays, we analyzed behavioral and metabolic data from Nt5c2 knockout (KO) mice available through the International Mouse Phenotyping Consortium (IMPC; *see Methods*). Among the available assays, several behavioral and metabolic parameters reached nominal significance (*p* < 0.05; Fig. 3A). We focused subsequent analyses on locomotor activity, acoustic startle/pre-pulse inhibition (PPI), and open field behavior, which yielded the most robust phenotypic alterations.

In the SHIRPA and dysmorphology battery, KO mice showed significantly reduced locomotor activity, measured as the number of squares crossed during the assay (Fig. 3B). A two-way ANOVA revealed a significant main effect of genotype [F (1, 5.33) = 9.45; *p* = 0.0211]. Sensorimotor gating was also affected. In the acoustic startle and PPI battery, KO mice displayed significantly reduced response amplitude to acoustic startle compared with WT [F (1, 32) = 11.86; *p* = 0.0016 for genotype; Fig. 3C]. While % pre-pulse inhibition (PPI1) also differed significantly, the effect was more variable (not shown). The reduction in startle amplitude was particularly evident in male mice. In the open field test, KO mice exhibited reduced exploratory behavior (Fig. 3D), reflected by fewer center entries (F (1, 1304) = 10.59; *p* = 0.0012 for genotype) and reduced center distance traveled [F (1, 1304) = 12.161; *p* = 0.0005 for genotype]. Several other anxiety-related measures were also altered, including % center time, latency to center entry, and center permanence (not shown). The anxiolytic-like phenotype was evident in both sexes. Together, these results indicate that Nt5c2 loss in mice impacts locomotion, sensorimotor gating, and anxiety-related behaviors. While the whole-body knockout design does not allow us to assign these phenotypes exclusively to neuronal mechanisms, the convergence of altered locomotion, stimuli response, and exploratory drive suggests partial overlap with the neuronal dNT5B-dependent phenotypes observed in *Drosophila*.

### NT5C2 variants are mostly associated with metabolic and neuro-psychiatric phenotypes

**Fig. 4:**
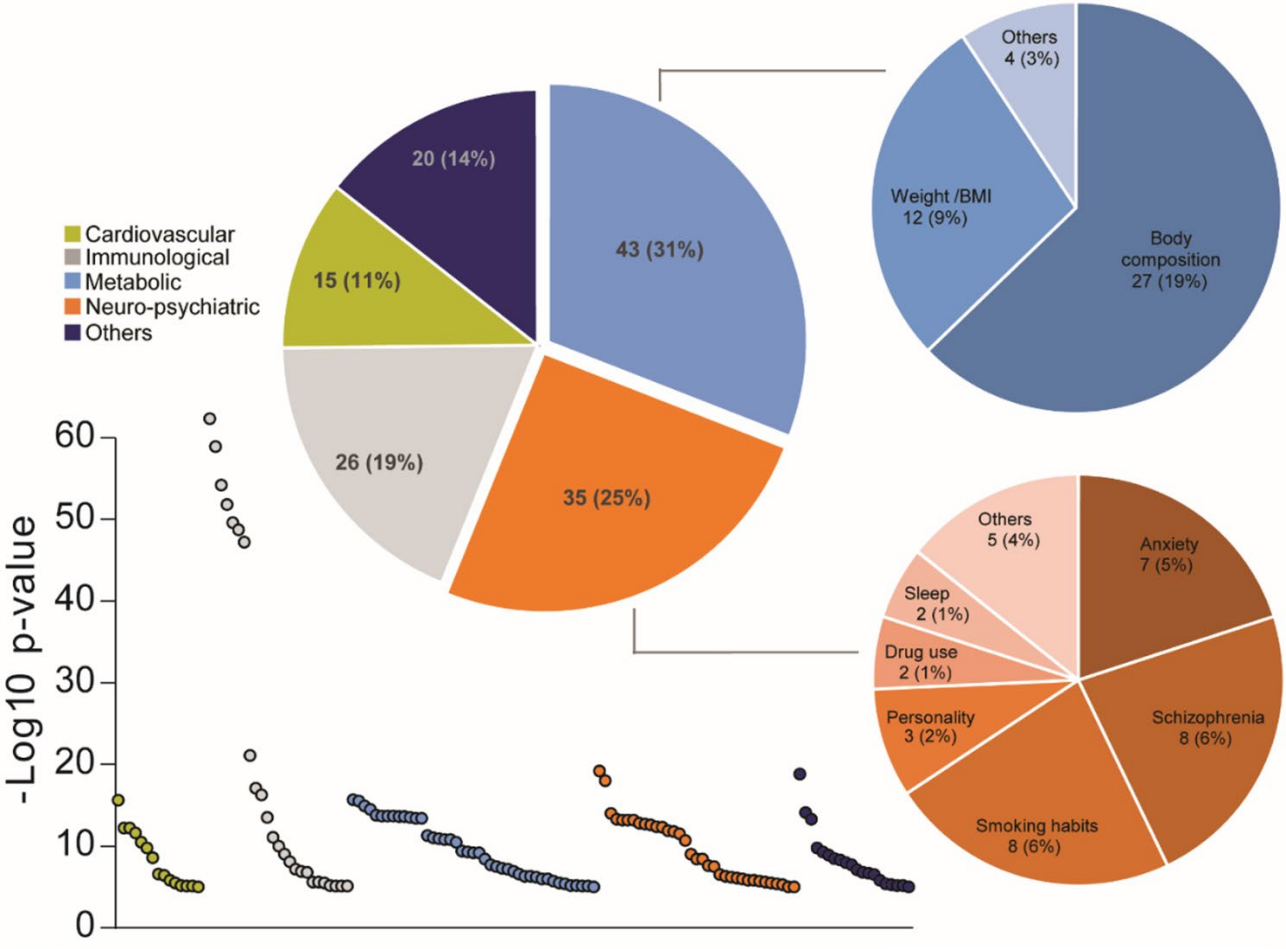
Phenome-wide association study (PheWAS) of NT5C2 variants reveals enrichment in metabolic and neuro-psychiatric traits. Scatterplot shows the significance of NT5C2 variant–trait associations (-log10 p-value) across different trait categories, with each point representing a significant association (corrected *p* < 0.05). Colors denote trait categories: cardiovascular (green), immunological (grey), metabolic (blue), neuro-psychiatric (orange), and others (dark blue). Pie charts summarize the distribution of significant associations by category, highlighting that metabolic (31%) and neuro-psychiatric (25%) traits predominate. Within metabolic associations, body composition (19%) and weight/BMI (9%) were most frequent. Within neuro-psychiatric associations, schizophrenia (6%), smoking habits (6%), and anxiety (5%) were most represented

Finally, to explore potential associations of NT5C2 with human traits, we queried the GWAS Atlas database and conducted a PheWAS analysis (*see Methods*). Significant variant-trait associations (*p* < 0.05 after correction) spanned multiple physiological domains, with the strongest signals clustering within metabolic and neuro-psychiatric categories (Fig. 4). Across all significant traits, 31% were classified as metabolic and 25% as neuro-psychiatric. Within the metabolic domain, the majority of associations related to body composition (19% of all traits) or weight/BMI measures (9%). In the neuro-psychiatric domain, top associations included schizophrenia (6%), smoking habits (6%), and anxiety (5%). Cardiovascular (11%), immunological (19%), and other categories (14%) comprised smaller fractions of the associations. These results indicate that common variants in NT5C2 show reproducible links to both metabolic and neuro-psychiatric phenotypes, with enrichment in traits related to body weight, body composition, and psychiatric conditions.

## Discussion

In this study, we combined *Drosophila* behavioral assays, mouse knockout data, and human genetic analyses to provide convergent evidence that NT5C2 plays a conserved role in modulating behavioral and metabolic traits. Our findings revealed that neuronal knockdown in *Drosophila* affected sleep-wake regulation and selectively suppressed feeding, while whole-body knockout in mice altered locomotion, sensorimotor gating, and exploratory drive. Genetic analyses in humans identified consistent associations of NT5C2 with body composition, BMI, and psychiatric traits, including schizophrenia and anxiety, supporting a conserved role across species and pointing to NT5C2 as a key link between metabolic balance and psychiatric vulnerability.

Specifically, in *Drosophila*, neuronal knockdown of dNT5B increased locomotor activity around light-dark transitions while reducing sleep fragmentation. Unlike previous reports that found either broad impairments in climbing [4] or only mild alterations in sleep and activity [8], our data highlight a more specific modulation of activity during environmental transitions. This discrepancy may reflect differences in behavioral paradigms and drivers, but also experimental conditions such as light intensity, which in our assays may have accentuated light-dark reactivity. Notably, our group has previously demonstrated a startle-like hyperlocomotor response to light-dark transitions, which is impaired in a *Drosophila* model of fragile X syndrome and enhanced in a schizophrenia model [42, 43]. It is intriguing that in the present study light-dark reactivity was also enhanced by neuronal dNT5B knockdown, while NT5C2 is linked to schizophrenia-associated phenotypes in mice and humans. However, whether this reflects a direct alteration of sensorimotor gating mechanisms in flies or a distinct circuit-level process remains to be determined.

We also extend prior work by examining feeding behavior in flies, where neuronal knockdown produced a clear reduction in food intake under satiated conditions, an effect absent during starvation. Importantly, although methodological constraints prevent us from fully excluding subtle effects of dNT5B knockdown under starvation, our sensitivity analysis indicates that large genotype-dependent differences are unlikely in this state. These findings suggest that dNT5B contributes to satiety-linked feeding regulation in addition to shaping reactions to environmental changes which influence the temporal structure of activity-rest cycles. Rather than broadly controlling appetite, dNT5B appears to act as a state-gated checkpoint that suppresses feeding when homeostatic drive is low, while leaving starvation-induced feeding intact. This architecture resembles mechanisms described in other conserved signaling pathways that gate feeding circuits and their dopaminergic interfaces [44, 45]. Such state-specific tuning may help explain how genetic variation can affect body weight over time without producing overt feeding phenotypes in acute assays. Our interpretation is that dNT5B/ NT5C2 dysfunction may shift the threshold for meal termination, providing a plausible explanation for BMI associations observed in human cohorts. Consistently, Nt5c2-deficient mice demonstrate resistance to diet-induced weight gain, with knockout animals gaining less fat on a high-fat diet and showing improved insulin sensitivity [26].

Analysis of IMPC data further supported conserved behavioral functions for NT5C2 beyond feeding behavior. Whole-body Nt5c2 knockout mice displayed reduced locomotion in the SHIRPA battery, decreased startle response amplitude and altered PPI, and robust differences in open field exploration. While these assays cannot disentangle central from peripheral effects, they mirror aspects of the *Drosophila* phenotypes, namely altered locomotor regulation and behavioral reactivity to environmental cues. Such convergence across models strengthens the interpretation that NT5C2 influences neuronal pathways shaping arousal, gating, and exploratory behaviors.

At the human level, we observed that PheWAS of NT5C2 variants highlighted associations spanning body weight, fat distribution, and glycemic measures, alongside psychiatric outcomes such as schizophrenia, anxiety, and smoking behavior. This dual pattern mirrors the cross-species findings and points toward a shared regulatory mechanism rather than independent effects on metabolism and mood. As suggested earlier, one possibility supported by animal data is that NT5C2 modulates satiety-dependent decision-making about food intake. In this scenario, genetic variation would not necessarily produce overtly measurable differences in eating habits on standard questionnaires (unless they reach clinical thresholds) but could still shift the likelihood of meal termination or initiation under satiety. Such subtle behavioral biases would accumulate over time, ultimately producing the BMI and body composition differences detectable in population cohorts. At the same time, the overlap with psychiatric traits indicates that this mechanism may intersect with neural circuits governing motivation and affect, providing a plausible basis for the pleiotropic risk architecture observed in genome-wide data.

An important caveat is that the cross-species approaches differ in genetic assessment (i.e. neuronal knockdown in flies, whole-body knockout in mice, and common variants in humans). This limits direct comparability, as phenotypes observed in the mouse model could arise from peripheral or developmental effects in addition to central mechanisms. As such, the mouse data should be viewed as complementary evidence for NT5C2 involvement in behavior, but not as definitive proof of a conserved neuronal role. Nevertheless, the convergence across flies, mice, and humans strengthens the interpretation that the observed behavioral alterations are not incidental but reflect a core property of NT5C2 function. Notably, both the animal data and human PheWAS point to overlapping domains, with associations involving Axis I psychiatric disorders or related behavioral traits, as well as reduced body mass.

Moreover, our feeding behavior results in flies highlight yet another potential mechanistic link, as neuronal dNT5B knockdown selectively reduced feeding under satiated conditions. Whether human body mass associations reflect subtle alterations in eating habits, likely below the detection threshold of standard GWAS questionnaires unless they reach clinical diagnosis or instead arise from additional non-neuronal energetic mechanisms remains unresolved. Further work will be required to disentangle these pathways and establish how NT5C2 integrates neurobehavioral regulation with systemic metabolic outcomes, potentially through AMPK–mTOR signaling, purinergic adenosine metabolism, or interactions with other psychiatric risk proteins.

Addressing these questions will require targeted experiments. For instance, feeding assays in Nt5c2 knockout mice could test for conserved satiety-dependent effects, while neuronal-specific knockouts would isolate central from peripheral contributions. In flies, replicating the feeding assays with alternative neuronal drivers would be valuable to confirm the robustness of the phenotype and to resolve discrepancies reported in previous studies. Additionally, a first step toward bridging behavior and molecular signaling would be to couple feeding paradigms with state-dependent measurements of AMPK pathway activity in neural tissue, quantifying phospho-AMPK, downstream mTOR readouts (e.g., phospho-S6K, 4E-BP), and AMP/ATP ratios in dissected brains collected after defined satiated versus starved or light–dark transition epochs. Complementary targeted metabolomics of purine-related metabolites (e.g., AMP, IMP, adenosine) in the same preparations would help establish NT5C2-dependent energetic effects and test whether dNT5B knockdown shifts the energetic set point at which satiety circuits adjust behavioral output. In parallel, experiments combining neuronal dNT5B knockdown with partial genetic or pharmacological modulation of AMPK-mTOR signaling could determine whether normalizing or further perturbing this pathway rescues or exacerbates the satiety-dependent feeding and light-dark reactivity phenotypes. Together, these approaches would provide molecular evidence in fly neural tissue that directly links NT5C2 disruption to altered behavioral regulation of energy balance.

## Conclusions

NT5C2 emerges as a conserved neuro-metabolic regulator that shapes context-dependent behaviors across species. Its reduction alters feeding under satiety, reorganizes activity-rest dynamics, and impacts locomotor and anxiety-related phenotypes, while human genetic data point to consistent links with psychiatric disorders and body mass regulation. Our findings suggest that NT5C2 does not act as a broad regulator of behavior or metabolism but instead fine-tunes specific processes that emerge under particular internal states (such as satiety) or external challenges (such as light-dark transitions). This state- and context-dependence provides a unifying framework to interpret its pleiotropic associations across flies, mice, and humans. Overall, these findings highlight NT5C2 as a gene at the intersection of energy balance and behavioral control, providing a plausible basis for its pleiotropic associations with both psychiatric vulnerability and metabolic traits.

## Data Availability

The datasets used and/or analyzed during the current study are available from the corresponding author on reasonable request.
